# Degradation of Three Herbicides and Effect on Bacterial Communities under Combined Pollution

**DOI:** 10.3390/toxics12080562

**Published:** 2024-08-01

**Authors:** Liangchi Mei, Xinle Xia, Jian Cao, Yuzhen Zhao, Haiyun Huang, Ying Li, Zhaoxian Zhang

**Affiliations:** Key Laboratory of Agri-Food Safety of Anhui Province, College of Resources and Environment, Anhui Agricultural University, Hefei 230036, Chinall15375337365@163.com (X.X.); 18005486603@163.com (Y.L.)

**Keywords:** herbicides, combined pollution, degradation behavior, metabolic pathway, microbial community

## Abstract

Pesticide residues in soil, especially multiple herbicide residues, cause a series of adverse effects on soil properties and microorganisms. In this work, the degradation of three herbicides and the effect on bacterial communities under combined pollution was investigated. The experimental results showed that the half-lives of acetochlor and prometryn significantly altered under combined exposure (5.02–11.17 d) as compared with those of individual exposure (4.70–6.87 d) in soil, suggesting that there was an antagonistic effect between the degradation of acetochlor and prometryn in soil. No remarkable variation in the degradation rate of atrazine with half-lives of 6.21–6.85 d was observed in different treatments, indicating that the degradation of atrazine was stable. 16S rRNA high-throughput sequencing results showed that the antagonistic effect of acetochlor and prometryn on the degradation rate under combined pollution was related to variation of the *Sphingomonas* and *Nocardioide*. Furthermore, the potential metabolic pathways of the three herbicides in soil were proposed and a new metabolite of acetochlor was preliminarily identified. The results of this work provide a guideline for the risk evaluation of combined pollution of the three herbicides with respect to their ecological effects in soil.

## 1. Introduction

Pesticides improve the quantity and quality of crops and their market shares reached 84.5 billion dollars in 2019 [1]. China is a large agricultural country, and its pesticide usage was the highest in the world from 2000 to 2019 according to a survey by the FAO. However, approximately 1% of pesticides reach target pests, and the remainder end up in the soil, water, and air, ultimately entering our food chain and affecting nontarget species, including humans, animals, and plants [2,3]. Chiaia-Hernandez et al. investigated 80 polar pesticides and >90 metabolites in archived topsoil samples from the Swiss Soil Monitoring Network (NABO) from 1995 to 2008, and the application patterns of the pesticides are known. The results showed that residues of approximately 80% of all applied pesticides could be detected, with half of these found as metabolites with a persistence of more than a decade [4].

Soil is the most important energy source in the world, and is the key to material storage and energy cycling on Earth [5]. Soils directly or indirectly affect many organisms. Therefore, crop quality and food safety are related to the quality of agricultural soil. Unlike air pollution and water pollution, soil pollution is always complex and difficult to measure. Soil is the most important part of the ecosystem and can be contaminated with pollutants, including antibiotics, pesticides, and heavy metals [6]. Pesticide residue in soil can cause a series of adverse effects on agricultural ecosystems by affecting the composition and diversity of microbial community, nitrogen transformation, beneficial organisms, and enzymes in soil [7]. Atrazine inhibits the activities of superoxide dismutase and catalase, and causes damage in earthworms [8]. Acetochlor in soil significantly alters fungal community structure and leads to an increase in pathogenic and noncultivatable fungal populations, which could increase the risk of plant disease outbreaks [9]. Boulahia et al. investigated whether *Phaseolus vulgaris* L. seedlings exposed to prometryn contaminated soil triggered an oxidative stress response and found that prometryn at concentration greater than 100 μM in soil inhibited bean growth and reduced the accumulation of photosynthetic pigments and photosynthetic products. Higher concentration prometryn (500 μM) has a disastrous effect, reducing antioxidant activities [10].

Compared with other agricultural chemicals, herbicides are characterized by greater production and usage [11]. By 2016, more than 2000 herbicides with 15 different action modes had been introduced into the global market. However, multiple herbicides are simultaneously applied during the crop growth cycle, leading to the complexity of pesticide residues in soil. There might be synergistic or antagonistic effects among different pesticides or between pesticides and other pollutants, making it difficult to estimate the ecological risk of pesticides to the soil [12]. Copper nanoparticles decreased the atrazine dissipation rate, and combined pollution did not alter the microbial communities in the soil [13]. Oxytetracycline (OTC) at an exposure concentration of 50 mg/kg could inhibit the degradation of atrazine in soil, increasing the environmental risk of the herbicides. Co-application of OTC and atrazine decreased the activities of soil urease, dehydrogenase, and catalase during short incubation periods [14]. Atrazine significantly stimulated soil cumulative net nitrogen mineralization and nitrification. However, the combination of atrazine and Pb had a significant inhibition effect on soil net nitrogen nitrification [15]. At present, studies on combined effect with multiple herbicides in environmental behavior and soil microorganisms are limited.

Acetochlor, prometryn, and atrazine are highly efficient and long-lasting pre-emergence herbicides, which are most commonly used in maize fields [16]. The repeated and large-scale application of these herbicides has resulted in widespread deposition in soils and surface water, accompanied by half-lives ranging from three to 57 weeks [17]. Acetochlor was applied at three different dosages of 2.11 L/h, 2.98 L/ha, and 3.80 L/ha, and the acetochlor concentrations at the 0–5 cm depth were 0.243, 0.301, and 0.304 mg/kg at 5 d [18]. The atrazine concentrations in the soil ranged from 0.01 to 0.2 μg/kg for subsoil and topsoil even 20 years after its ban [19]. The three herbicides possibly pose an environmental risk, including the contamination of arable soil, surface water, and groundwater, due to their persistence in the environment. Furthermore, acetochlor, prometryn and atrazine are endocrine-disrupting chemicals and probable human carcinogens that can induce endocrine disruption, oxidative stress, immunotoxicity, or reproductive-toxicity in nontarget organisms [20,21].

In this work, a reliable and efficient gas chromatography–mass spectrometry (GC–MS) method for the simultaneous determination of these three herbicides in soil was established to investigate the interactive effects on their degradation rates. The microbial assemblages were profiled by 16S rRNA high-throughput sequencing under the combined exposure conditions to reveal the interactive effect mechanism. Furthermore, the metabolic pathways of the three herbicides were proposed using liquid chromatography-time-of-flight/mass spectrometry (LC-TOF/MS). The results provide a reference for the soil risk evaluation of combined pesticide pollution on soil ecological health.

## 2. Experimental Section

### 2.1. Materials and Reagents

Acetochlor, prometryn, and atrazine standards were obtained from Hefei Baierdi Chemical Technology Co., Ltd. (Hefei, China) with purities of 98.5%, 99.4%, and 99.52%, respectively. HPLC-grade acetonitrile was obtained from Sigma Aldrich Trading Co., Ltd. (Shanghai, China). Sodium chloride (NaCl) was acquired from TITAN Technology Co., Ltd. (Shanghai, China). Acetone was purchased from Xilong Chemical Co., Ltd. (Guangzhou, China). Ultrapure water was prepared using a UPT-11-10T water purification system. All other chemicals were of analytical grade and were purchased from commercial sources.

### 2.2. Incubation of Soils

The experimental soil was collected from the top 15 cm of soil in Dangshan County, Anhui Province, China. The collected soil was air-dried and sieved through a 2-mm mesh. The physical and chemical characteristics of the soil are shown in Table S1. The soil was determined using LC-MG/MS to confirm that there were no endogenous distractors. The soil was preincubated by adding an appropriate amount of water for 7 d at 30 °C in the dark to activate the soil microorganisms. Then, incubation experiments with acetochlor, prometryn, and atrazine, individually and in combination, were conducted in soils. A standard solution of 3.5 mL of the herbicides with a concentration of 10,000 mg/kg to 7 kg of soil was air-dried for 30 min [22,23]. The initial concentration of the herbicides in soil was 5 mg/kg. The specific treatment conditions in triplicate were as follows: Group CK (Control, natural soil without the herbicides); Group T(A) (acetochlor); Group T(P) (prometryn); Group T(AT) (atrazine); Group T(A+P) (acetochlor and prometryn); Group T(A+AT) (acetochlor and atrazine); Group T(P+AT) (prometryn and atrazine); and Group T(A+P+AT) (acetochlor, prometryn, and atrazine). Purified water was added to the soil samples to adjust the water content, and the final water content was set as approximately 60% of the saturated water holding capacity (*w*/*w*). The soil samples were incubated under normal sunlight at room temperature (25 °C). During incubation experiments, the soil samples were regularly weighed, and purified water was added to maintain moisture stability. Ten-gram soil samples were collected at 0, 1, 3, 5, 7, 14, 28, 50, 70, and 100 d to determine the residues of herbicides and their metabolites.

### 2.3. Pretreatment Method

Two grams of soil from the collected soil samples at regular time intervals was accurately weighed and transferred to a 50 mL polyethylene centrifuge tube. Then, 5 mL of water, 2 g of sodium chloride, and 10 mL of acetonitrile were added. The mixture was shaken violently for 3 min, followed by ultrasonic extraction for 3 min. Next, the sample was centrifuged for 5 min at 4000 rpm. Five milliliters of supernatant was collected and dried using nitrogen. The residue was dissolved with 1 mL acetone and then filtered through a 0.22 µm nylon syringe filter for GC–MS analysis.

### 2.4. Analysis Method

A sensitive and reliable quantitative method was established to simultaneously determine three herbicides using Agilent 7890A-7000C (Agilent Technologies, Palo Alto, CA, USA) with an HP-5MS quartz capillary column (30 m × 0.25 mm i.d., 0.25 μm). The sample was analyzed with electron impact (EI) mode, and the MS system was programmed in scan mode ranging from 50–600 Da. The interface temperature was adjusted to 280 °C, and the ion source temperature was set to 180 °C. The carrier gas (He) flow rate was set at 1 mL/min without splitting. The injection volume and inlet temperature were set at 5 µL and 280 °C, respectively. The column temperature gradient was as follows: 60 °C initial (1 min), increased to 170 °C at 40 °C/min (0 min hold), and increased to 260 °C at 10 °C/min (3 min hold).

### 2.5. Method Validation

The quality assurance and quality control (QA/QC) metrics for the pesticide analysis consisted of specificity, linearity, limit of detection (LOD), limit of quantification (LOQ), precision, and accuracy. Blank samples were extracted according to the above pretreatment method and analyzed to confirm the specificity of the method. The linearity of the solvent and matrix calibration curves was evaluated at concentrations of 0.05, 0.1, 0.5, 1, and 5 mg/L in triplicate. The slope ratio of the solvent and matrix calibration curve was used to determine the matrix effect. The matrix-dependent LODs and LOQs of the herbicides in the soil samples were determined as the lowest concentration that produced a signal-to-noise (*S*/*N*) ratio of 3 and 10, respectively. The concentrations corresponding to the *S*/*N* of 3- and 10-times peak areas were LOD and LOQ, respectively.

Recovery experiments were carried out to evaluate the accuracy and precision of the pretreatment method. Blank soil was spiked with standard solutions of herbicides at different concentrations in quintuplicate and the final concentrations of the target compound in the soil were 0.1, 1, and 5 mg/kg. Then, the soil samples were extracted using the above pretreatment procedure. The accuracy and precision of the method were evaluated with recoveries and relative standard deviations (RSDs), respectively.

### 2.6. Metabolite Determination

Soil samples (20.0 g) exposed to the three herbicides for 50 d were extracted according to the above pretreatment method. The final residue was dissolved in 1 mL acetonitrile. The mixture was filtered through a 0.22 µm nylon syringe filter for LC-TOF/MS analysis (AB SCIEX, MA, USA) with a C_18_ column (2.1 × 50 mm i.d., 2.7 μm). The LC-TOF/MS parameters are described in the supplementary material. The test data were analyzed using SCIEX OS-Q (Version 2.1), and MetabolitePilot™ (Version 2.0) software. The software produced a series of metabolites, then the structure of these metabolites was further examined by analyzing the chromatographic peaks, fragmentation patterns, characteristic isotopic peaks, and mass error of the metabolites to eliminate insignificant and impossible results. The following criteria were used to filter the potential metabolites: (1) chromatographic peaks were detected in the treatment samples but not in the control; (2) the peak area showed an increasing trend or the peak area initially increased and subsequently decreased with the extension of the incubation time; (3) satisfactory peak shape; (4) the mass error of precursor ion <±5 ppm; (5) characteristic isotopic peaks; and (6) >2 matched common fragments.

### 2.7. Analysis of Soil Microbial Community Structure

The total DNA of the soil samples at 0, 28, 70, and 100 d was extracted using the MoBioPowerSoil™ DNA Isolation Kit (Mo Bio Laboratories Inc., Carlsbad, CA, USA) according to the manufacturer’s instructions. The concentration and quality of the extracted DNA were determined using a NanoDrop 2000 (Thermo Fisher Scientific, Cleveland, OH, USA). The V3 and V4 gions of the 16S rRNA gene were amplified with region-specific primers (338F/806R). The PCR test used TransStart Fastpfu DNA polymerase (AP221-02, TransGen, Beijing, China) and a 20 μL reaction system (5 × FastPfu buffer 4 μL, 2.5 mM dNTPs 2 μL, 5 μM forward primer 0.8 μL, 5 mΜ reverse primer 0.8 μL, FastPfu polymerase 0.4 μL, BSA 0.2 μL, template DNA 10 ng, and balanced to 20 μL with ddH_2_O). The PCR amplification products were tested by gel electrophoresis (2%). The samples were sequenced using the MiSeq Illuminate PE300 platform at Shanghai Majorbio Bio-pharm Technology Co., Ltd (Shanghai, China).

### 2.8. Data Analysis

The degradation rate constants (k) of the three herbicides were determined using the first-order kinetic equation:

Ct = C_0_e^−kt^

where C_0_ is the initial concentration in the soil and Ct is the concentration at time t. The half-life (T_1/2_) was calculated with the following equation:

T_1/2_ = ln2/k

A normalized number of sequences was randomly extracted from each sample to calculate the α diversity indices, which were estimated with the “vegan” package in R. Nonparametric t tests were used for detection of significance for the Shannon diversity, Pielou evenness, and Chao1 index in R with the “EasyStat” package. Before the calculation of *beta* diversity, relative abundances were used to standardize the OTU profiles. Bray-Curtis similarity matrices were prepared using the “vegan” package in R. Permutational multivariate analysis of variance (PERMANOVA) (adonis, data transformed by Bray-Curtis, permutations = 999) was used to test whether the β diversity differed among treatments. Then, principal coordinate analysis (PCoA) plots or t-distributed stochastic neighbor embedding (t-SNE) plots were generated according to the Bray-Curtis similarity matrices created using the R package “ggplot2”. t-SNE analysis was performed by the R package “t-sne”. Network analysis was performed using the R package “ggClusterNet” on GitHub.

## 3. Results and Discussion

### 3.1. Degradation of Three Herbicides in Soil

**Figure 1:**
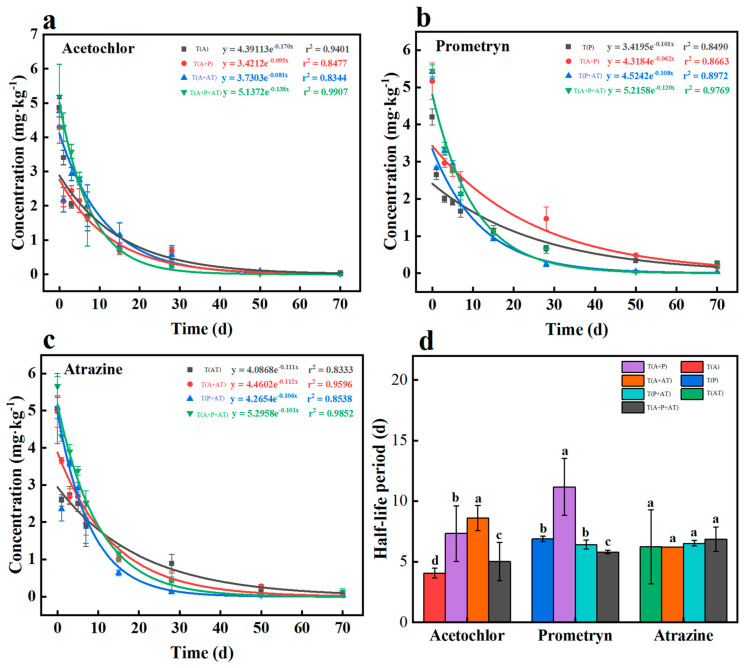
The degradation of the acetochlor (**a**), prometryn (**b**), and atrazine (**c**) in soil, (**d**) The half-life of three herbicides in different treatments.

To investigate the interactive effects of the three herbicides on their degradation rates, soil incubation was conducted under individual and combined exposure conditions using the satisfactory method of pretreatment and determination (Table 1 and Table S2 and Figure S1). The method validation is described in Supplementary Materials. As shown in Figure 1, the initial concentrations of acetochlor, prometryn, and atrazine in the different treatments were 4.7–5.0 mg/kg, 4.7–5.2 mg/kg, and 4.9–5.3 mg/kg, indicating that the three herbicides were uniformly distributed in soils with different treatments. The degradation of the three herbicides was well described via a first-order kinetic equation with decisive coefficient (R^2^) values of 0.8344–0.9907. After the 70-d soil incubation experiment, more than 90% of the herbicides were degraded. The half-lives of acetochlor, prometryn, and atrazine were 4.1 d, 6.9 d, and 6.2 d, respectively, in the individual exposure experiments. The reported half-life of acetochlor in the field ranges from 3.4–29 d, with a mean value of 12.9 d [24,25]; the half-life of prometryn was 2–43 d in soils [26]; and the half-life of atrazine ranged from 4 to 57 weeks [27]. There were significant differences between the reports concerning the half-life of the three herbicides. Based on previous reports, the reasons for the differences might be due to (1) different initial concentrations affecting the degradation rate of the herbicides in soil, (2) soil physical and chemical properties including pH, organic matter content, temperature, cation exchange capacity and etc., affecting the degradation rate of the herbicides by affecting existential state and soil microbial activities [28], and (3) the diversity and composition of soil microorganisms [29].

Under combined exposure (binary or ternary mixtures), the degradation rates of acetochlor and prometryn were inhibited to different degrees. In the binary exposure experiment, the half-lives of acetochlor in T(A+P) and T(A+AT) were 1.8 and 2.1 times that of individual exposure, implying that prometryn and atrazine could effectively inhibit the degradation of acetochlor in soil. Also, the inhibiting effect of atrazine on the degradation of acetochlor was higher than prometryn. There was no remarkable difference in the degradation rate of prometryn in the presence of atrazine compared with individual exposure, which means that atrazine did not affect the degradation of prometryn. However, acetochlor showed a significant inhibitory effect on the degradation rate of prometryn. The half-life of prometryn was increased to 1.6 times that of individual exposure. Compared to prometryn and acetochlor, the degradation rate of atrazine in soil was stable in individual and combined exposure experiments, and the half-lives were 6.21–6.85 d. Swarcewicz et al. investigated the combined effects of multiple pesticides on the degradation of pendimethalin in soils, and the results showed that the degradation of pendimethalin was significantly inhibited by mancozeb and thiamethoxam [30]. The above results indicated that the combined pollution of multiple pesticides might result in greater herbicide persistence, leading to an increased risk of environmental contamination. In fact, there are many factors that affect the interactions of pesticides in soil, particularly the microbial population. Therefore, we further explored the combined effects of the three herbicides on soil microorganisms to explore the biomechanism of herbicide interactions.

### 3.2. Changes in Soil Bacterial Community Structure

**Figure 2:**
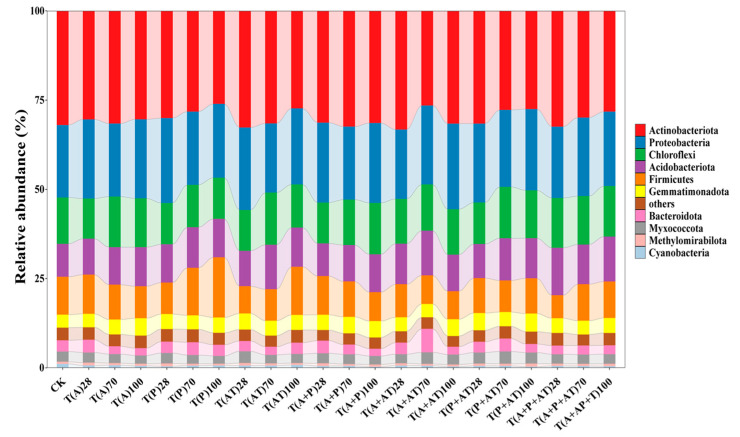
Phylum-level distribution of the sandy soil samples at 28 d, 70 d, 100d. CK means control groups.

In total, 3,923,555 high-quality sequences were obtained across a total of 67 soil samples from 23 treatments consisting of three replicates each. The average read count of each sample was 59448 (standard deviation (SD) 6709). The optimized sequences were clustered into approximately 12,317 OTUs under 100% similarity. The rarefaction curves showed that the sequencing data were basically reasonable, and no notable contribution to the discovery of new OTUs was observed with an increase in the amount of data (Figure S2). The majority of OTUs belonged to the phyla *Actinobacteriota* (30.19%), *Proteobacteria* (21.55%), *Chloroflexi* (12.88%), *Acidobacteriota* (10.94%), *Firmicutes* (9.93%), *Gemmatimonadota* (4.27%), *Bacteroidota* (2.99%), *Myxococcota* (2.76%), *Methylomirabilota* (0.61%) and *Cyanobacteria* (0.57%) (Figure 2).

There was no remarkable significant difference in the alpha diversity indices, consisting of richness, Shannon diversity, and Pielou’s evenness under different exposure conditions (Figure S3). Ordination using PCoA, based on Bray-Curtis distances, illustrated a significant (adonis, *p* = 0.001, PERMANOVA) difference among the treatments (Figure S4). In particular, the composition of microorganisms showed a remarkable difference under individual exposure to the three herbicides. However, the difference in the composition of microbial species was not remarkable with combined exposure, suggesting that the interaction of the three herbicides potentially mitigate the adverse effects on soil ecology health.

**Figure 3:**
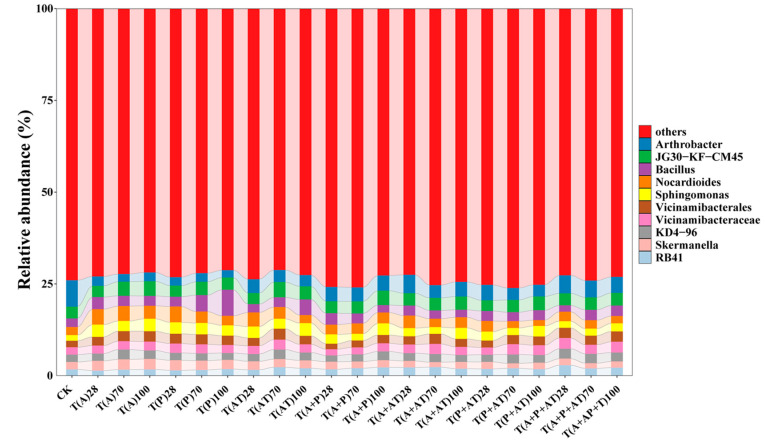
Genus-level distribution of the sandy soil samples at 28 d, 70 d, 100 d. CK means control groups.

The composition of microorganisms in the samples at the genus level was analyzed. A total of 10 major genera including *Arthrobacter* (3.63%), *JG30-KF-CM45* (3.45%), *Bacillus* (2.90%), *Nocardioides* (2.88%), *Sphingomonas* (2.60%), *Vicinamibacterales* (2.39%), *Vicinamibacteraceae* (2.21%), *KD4-96* (2.21%), *Skermanella* (2.05%), and *RB41* (1.94%) were observed (Figure 3). The abundance of Arthrobacter under combined exposure to the herbicides was significantly increased compared to that under individual exposure. The abundance of *Arthrobacter* under combined exposure increased by 51%, 94%, and 91% in Group D, Group E, and Group G, respectively, compared with that of an individual exposure to acetochlor at 28 d. Compared with exposure to prometryn, the abundance of *Arthrobacter* increased by 69%, 82%, and 112% at 28 d. The abundance of *Arthrobacter* increased by 33%, 12%, and 30% at 28 d compared with individual atrazine exposure. The abundance of *RB41* exhibited an increasing trend ranging from 20% to 104% under combined exposure. However, the abundance of *Nocardioides* decreased by 11% to 40% under combined exposure to the three herbicides compared with individual exposure. Previous studies have shown that *Sphingomonas* can effectively degrade acetochlor in soil [31]. Compared with acetochlor individual exposure, the abundance of *Sphingomonas* was significantly reduced under combined exposure conditions, leading to a lower degradation rate of acetochlor. Additionally, the sequencing analysis showed that the inhibitory rate of atrazine (33%) on *Sphingomonas* was higher than that of prometryn (24%), which indicated that the high inhibition rate of atrazine to acetochlor degradation may be due to its higher inhibition against *Sphingomonas*. This phenomenon was consistent with the results of the degradation experiments. These findings implied that prometryn and atrazine may inhibit the degradation of acetochlor in soil by inhibiting the abundance of *Sphingomonas*. Satsuma isolated the bacterial strain *Nocardioides* sp. DN36 from s-triazine polluted sites and found that the strain could efficiently degrade prometryn and its methylthio-s-metabolite [32]. In this work, the abundance of *Nocardioides* was decreased in the presence of acetochlor compared with prometryn individual exposure. Therefore, acetochlor may inhibit the degradation rate of prometryn in soil by affecting *Nocardioides*.

**Figure 4:**
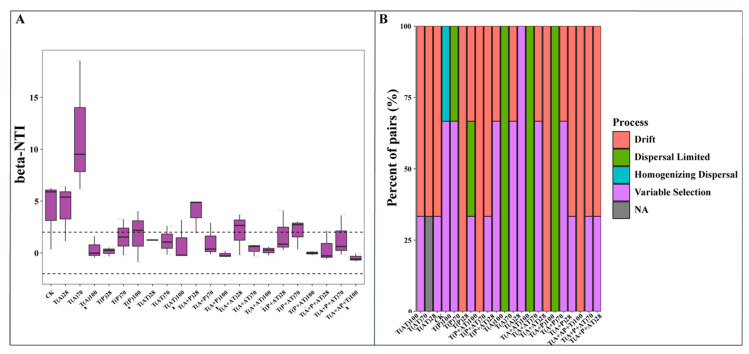
Deterministic and stochastic processes in microbiome assembly at 28, 70, and 100 d. (**A**): The deterministic and stochastic on microbiome assembly along different soil samples based on the β-Nearest Taxon Idex (βNTI) values. The βNTI values were calculated using null model, and |βNTI| ≥ 2 and |βNTI| < 2 represent dominant determinism and stochasticity in driving microbiome assembly, respectively. (**B**): based on the Raup-Crick index of Bray–Curtis (RCbray), deterministic and stochastic processes are divided into five basic ecological processes, including drift, dispersal limited, homogenizing dispersal, variable selection, and NA. The percentage accumulation map shows the relative importance of five ecological processes in the different treatment groups.

After calculation of the β-nearest taxon index (βNTI) between sample pairs, variable selection was more easily observed under combined exposure than individual exposure. The abundant community composition was determined by both deterministic (39.16%) and stochastic processes (60.83%). The latter consisted of homogeneous diffusion (17.34%), limited diffusion (4.76%), and nondominant processes (Figure 4).

### 3.3. Metabolic Pathways of the Herbicides in Soil

**Figure 5:**
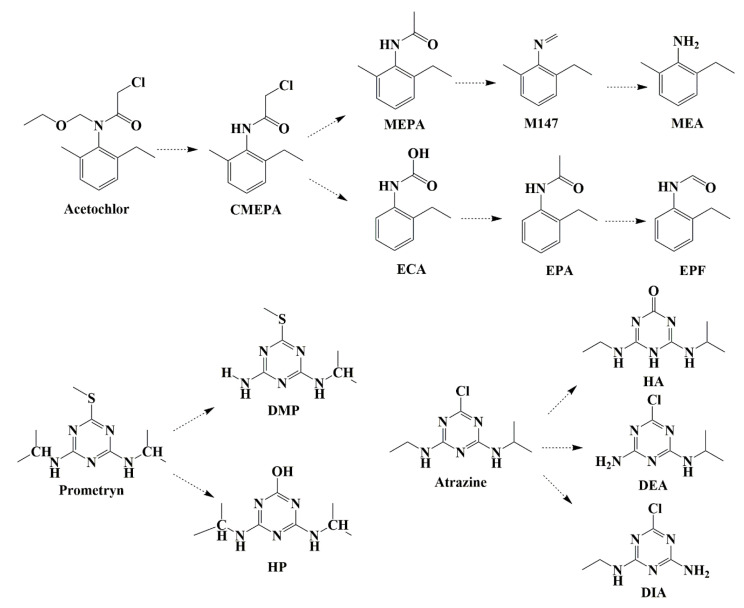
Potential degradation pathways of three herbicides in soil.

The degradation of pesticides in soil is mainly driven by hydrolysis, photolysis, and microbial degradation. Xu et al. isolated a bacterial strain of *Pseudomonas oleovorans* that degraded the chloroacetamide herbicide acetochlor and defined the degradation pathway of acetochlor, which included dechlorination, hydroxylation, *N*-dealkylation, *C*-dealkylation, and dehydrogenation [33]. The metabolites of acetochlor in the soil mainly included CMEPA, ECA, EMEMA, EPA, EPF, MEA, and MEPA. The degradation pathway of prometryn in soil was mainly driven by hydroxylation, dealkylation, and demethylation of demethylthio groups, and the degradation metabolites were DMP, DSP, DSPP, DPP, HDMP, DDPP, HP, HDPP, and HDDPP [34]. The degradation products of atrazine in soil mainly include hydroxylation atrazine (HA), deethylation-atrazine (DEA), deisopropylation-atrazine (DIA), deisopropylhydroxyl-atrazine (DIHA), and deethylation- and deisopropylation-atrazine (DDA) [35]. To investigate the degradation pathway of the three herbicides, soil incubation experiments with exposure to the three pesticides were carried out. As shown in Table 2 and Figure 5, the potential metabolic pathways of the three herbicides were proposed. Seven metabolites were observed with the degradation of acetochlor in the soil. A new degradation product of acetochlor, M147, was found in the soil. Only two metabolites of prometryn, the hydroxyl product (HP) and deisopropyl product (DMP) were identified in the soil. The secondary fragment ions of the parent compounds and metabolites are shown in Figures S5–S19. Khan et al. used GC and GC/MS methods to detect prometryn in field-treated soil and found that the metabolites HP and DMP were the only extractable degradation products in the field samples [36]. Our results were consistent with those previously reported. For atrazine, there were three metabolites, HA, DEA, and DIA in the soil.

## 4. Conclusions

In conclusion, there was a remarkable interactive effect among the three herbicides on their degradation and the bacterial community in the soil. There was a significant antagonistic effect between the degradation of acetochlor and prometryn in soil, leading to an increase in the environmental risk of the two herbicides. The antagonistic effect was related to variation of *Sphingomonas* and *Nocardioide*. The results do not recommend simultaneous application of both herbicides. In addition, we should consider other pollutants existing in the soil when evaluating the ecological risk of pesticides in the soil, rather than evaluating only the applied pesticides themselves.

## Figures and Tables

**Table 1:** The calibration curve, the limits of detection (LODs), limits of quantification (LOQs) of the three herbicides.

Herbicide	Substrate	Regression Equation	*R* ^2^	Slope Ratio	LOD (mg/kg)	LOQ (mg/kg)
Acetochlor	solvent	y = 685751x − 194712	0.9902	-	0.02	0.07
	soil	y = 794713x + 81646	0.9974	1.16	0.01	0.03
Prometryn	solvent	y = 1180609x − 375880	0.9905	-	0.002	0.005
	soil	y = 1941626x + 214145	0.9966	1.64	0.0003	0.001
Atrazine	solvent	y = 1319949x − 402477	0.9901	-	0.01	0.03
	soil	y = 1598530x + 103529	0.9982	1.21	0.001	0.003

**Table 2:** Summary of mass spectrometric data for metabolites and conjugates of the three herbicides.

Number	Compound	Chemical Formula	*t*_R_ (min)	*m*/*z*, [M + H]^+^	△ppm	MS^2^ Main Fragments (*m*/*z*)
1	Acetochlor	C_14_H_20_ClNO_2_	13.44	270.0946	-	270.0946, 224.0822, 206.0740, 148.1118, 133.0886, 90.0115, 59.0515
2	CMEPA	C_11_H_14_ClNO	0.76	212.1183	−0.4	212.1181, 195.0914, 119.0690, 94.0659, 77.0404
3	MEPA	C_11_H_15_NO	9.53	178.1222	−2.6	178.1637, 133.0656, 105.0699, 103.0558, 90.0394
4	M147	C_10_H_13_N	13.50	148.1114	−4.3	148.1135, 133.0892, 132.0807, 117.0578, 115.0549
5	MEA	C_9_H_13_N	0.74	136.1136	2.4	136.1136, 121.0886, 106.0659, 79.0358
6	ECA	C_9_H_11_NO_2_	0.75	166.1222	−2.9	161.1213, 150.0905, 136.0715, 109.0522, 96.0449
7	EPA	C_10_H_13_NO	0.73	164.1422	−2.6	164.1442, 148.1064, 133.1043, 120.0777, 104.8918
8	EPF	C_9_H_11_NO	0.73	150.1253	3.2	150.1253, 133.0478, 108.9271, 64.9381, 42.0385
9	Prometryn	C_10_H_19_N_5_S	9.12	242.1434	-	242.1434, 200.0962, 158.0494, 116.0281, 110.0471, 110.0780, 85.0525, 68.0268
10	DMP	C_7_H_13_N_5_S	8.84	200.0955	−4.7	200.0967, 158.0476, 110.0483, 85.0495, 68.0241
11	HP	C_9_H_17_N_5_O	4.49	212.1500	−2.7	212.1501, 170.1031, 128.0564, 119.0601, 86.0356, 77.0396, 69.0097
12	Atrazine	C_8_H_14_ClN_5_	9.32	216.1015	-	216.1015, 174.0554, 146.0225, 132.0324, 110.0459, 104.0012, 96.0562, 79.0069, 71.0618, 68.0260
13	DIA	C_5_H_8_ClN_5_	0.95	174.0535	−3.3	174.0535, 146.0234, 138.0791, 110.0458, 104.0017, 96.0564, 79.0077, 68.0263, 61.9809
14	DEA	C_6_H_10_ClN_5_	4.08	188.0690	−4.0	188.0700, 146.0223, 110.0484, 104.0003, 79.0054
15	HA	C_8_H_15_N_5_O	0.76	198.1346	−1.7	198.1346, 156.0882, 114.0664,97.0402, 86.0359, 71.0621, 69.0103

## Data Availability

The data will be provided if required.

## Supplementary Materials

The following supporting information can be downloaded at: https://www.mdpi.com/article/10.3390/toxics12080562/s1, LC-TOF/MS Method; Table S1: The physical and chemical characteristics of the soil; Table S2: Precision and accuracy of the GC/MS analysis method; Figure S1: typical chromatograms of standard liquid of three herbicides and blank matrixes; Figure S2: The rare curves of OTUs at 28, 70 and 100 d. Y means acetochlor, P means prometryn and A means atrazine; Figure S3: Alpha diversity of microbial community at 28, 70 and 100 d. The Shannon index (a), Richness index (b) and Pielou eveness (c) of samples; Figure S4: Beta diversity of microbial community explained by PCoA at 0, 28, 70 and 100 d; Figure S5–S19: Secondary fragment ion of acetochlor, CMEPA, MEPA, M147, MEA, ECA, EPA, EPF, prometryn, DMP, HP, atrazine, DIA, DEA and HA.
