# Supplementary material for: Degradation of Three Herbicides and Effect on Bacterial Communities under Combined Pollution
Source: Toxics. 2024 Aug 1;12(8):562. doi: 10.3390/toxics12080562 (PMC11360099; doi:10.3390/toxics12080562)
Supplement: Supplementary file 1 [file toxics-12-00562-s001.zip › toxics-3076904-supplementary.pdf]

Supplementary Materials

Degradation of Three Herbicides and Effect on Bacterial Communities under Combined Pollution

Liangchi Mei, Xinle Xia, Jian Cao, Yuzhen Zhao, Haiyun Huang, Ying Li and Zhaoxian Zhang *

Key Laboratory of Agri-Food Safety of Anhui Province, College of Resources and Environment, Anhui Agricultural University, Hefei 230036, China; ll15375337365@163.com (X.X.); 18005486603@163.com (Y.L.)

* Correspondence: zhangzx@ahau.edu.cn; Tel.: +86-188-0158-7786

LC-TOF/MS Method. The LC parameter settings were as follows: injection volume, 20 μL; column temperature, 30 ℃; flow rate, 0.3 mL min^{-1}. A gradient procedure with acetonitrile and water was carried out to better achieve the separation of metabolites. The details were as follows: 20−90% acetonitrile from 1−22 min, 90% acetonitrile for 3 min and then returned to the initial conditions for 5 min. The water included 0.1% formic acid. The MS parameter settings were as follows: the collision energies were 10 V and 40 V; the declustering potential was 80 V; the collision energy spread was 20 V; both ion source gases 1 and 2 were 65 psi; the curtain gas was 35 psi; the ion source temperature was 550 °C; the ion spray floating voltage was 5500 V; the ion release delay was 67 V; and the ion release width was 25 V. The scanning range was set to 20-1500 Da. The samples were analyzed in both positive and negative ion modes.

Method Validation. GC–MS method was established to simultaneously separate and determine the three herbicides within 15 min, and the retention times of acetochlor, prometryn and atrazine were 9.813 min, 10.015 min and 8.169 min, respectively (Figure S1). Then, blank soil samples were measured, and there was no impurity interference near the retention times of the target compounds, which means that the analysis method was specific. Good linearities of the solvent and matrix standard curves were observed with correlation coefficient (R^2) values of 0.9901-0.9982 (Table 1). The slope ratio was 1.16-1.64, suggesting that there was a remarkable matrix enhancement effect. Therefore, a matrix standard curve was prepared to quantitatively study the three herbicides in the soil. The LODs and LOQs of the three herbicides were 0.3-20 μg/kg and 1.0-70 μg/kg (Table 1), respectively. The average recoveries of the three herbicides in soil were 71%-87% with RSDs of 1.3-8.6% (Table S1), which means that the precision and accuracy of the method were satisfactory. Therefore, the analysis method meets the requirements for the residue detection of the three herbicides in soil.

Table S1. The physical and chemical characteristics of the soil.

Soil	TOC (g/100g)	Total N (g/100g)	pH	$cmol^{+}/kg$	sand（%）	silt（%）	clay（%）
Sandy soil	1.252	0.041	8.42	7.056	90.63	8.76	0.61

Table S2. Precision and accuracy of the GC/MS analysis method.

Herbicide	Spiked concentration (mg kg^{-1})	Average recovery rate (%)	RSD (%)
Acetochlor	0.1	79	8.6
	1	71	1.3
	5	72	3.2
Prometryn	0.1	76	7.4
	1	84	6.0
	5	87	4.3
Atrazine	0.1	77	7.9
	1	76	8.6
	5	81	3.8

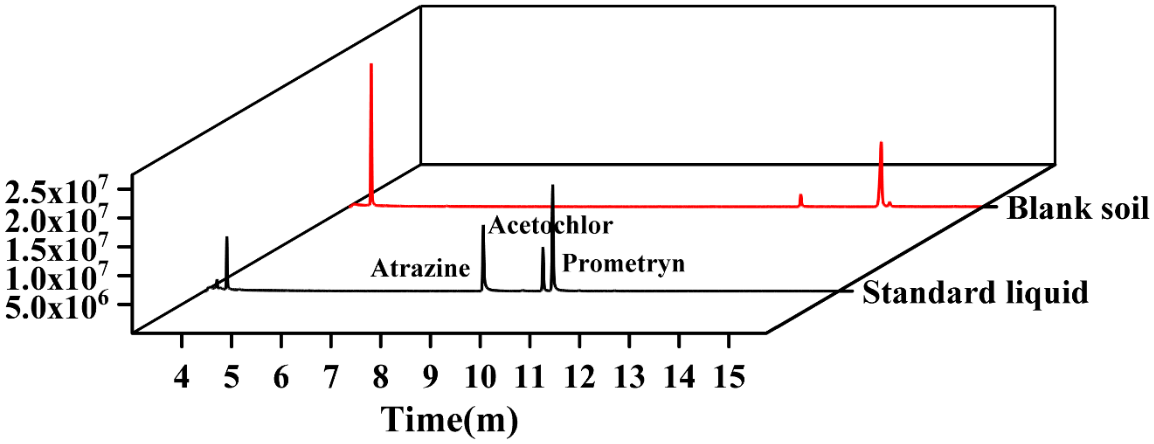

Figure S1. The typical chromatograms of standard liquid of three herbicides and blank matrixes.

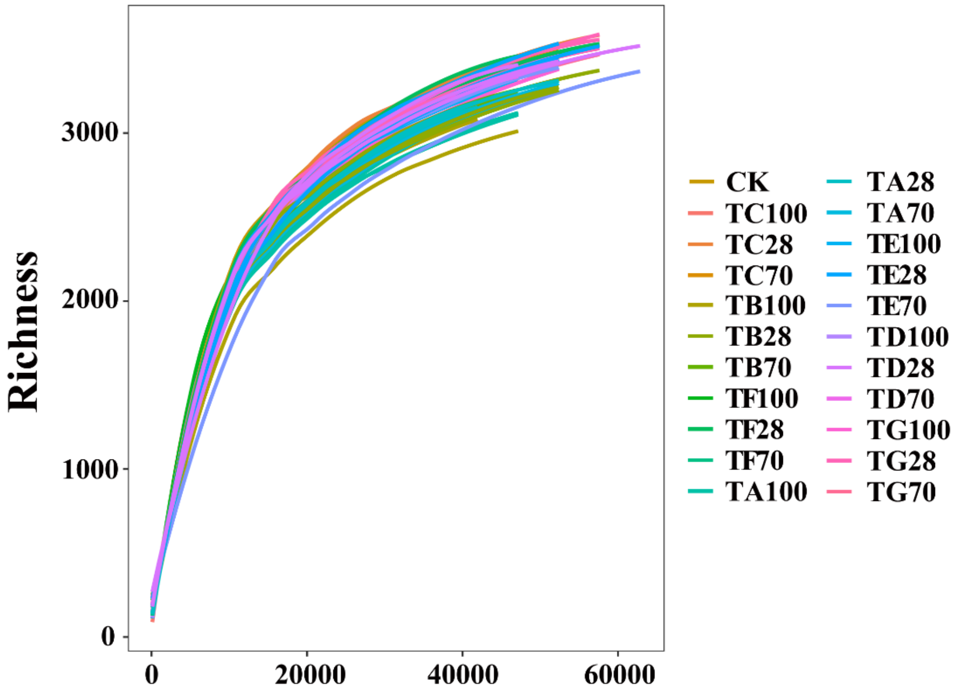

Figure S2. The rare curves of OTUs at 28, 70 and 100 d. Y means acetochlor, P means prometryn and A means atrazine.

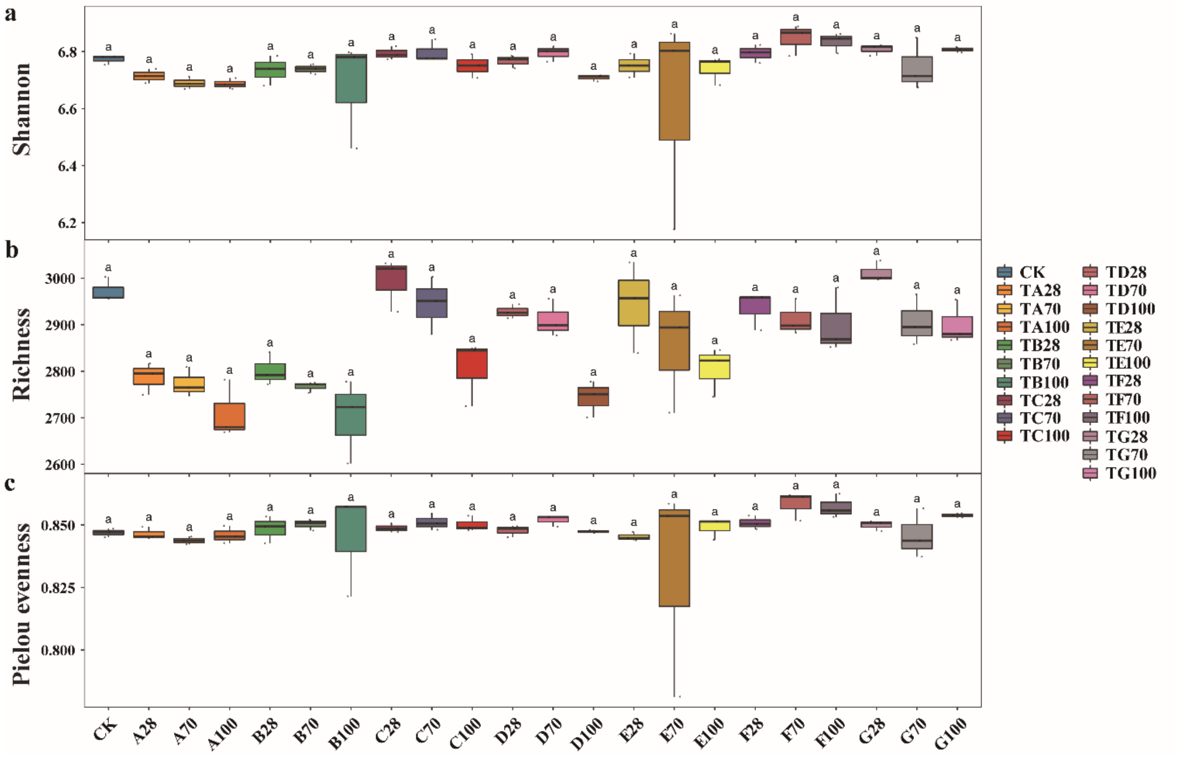

Figure S3. Alpha diversity of microbial community at 28, 70 and 100 d. The Shannon index (a), Richness index (b) and Pielou eveness (c) of samples.

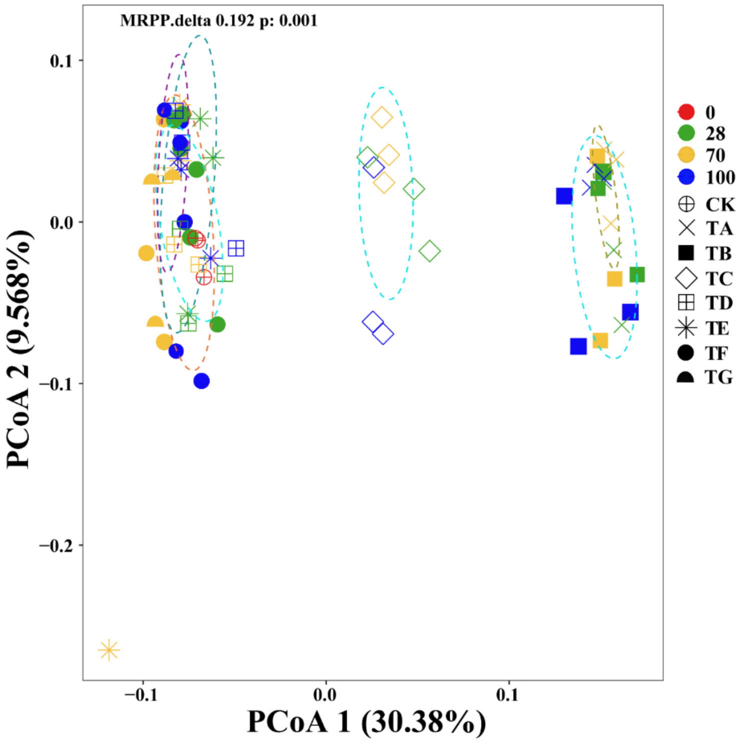

Fig. S4. Beta diversity of microbial community explained by PCoA at 0, 28, 70 and 100 d.

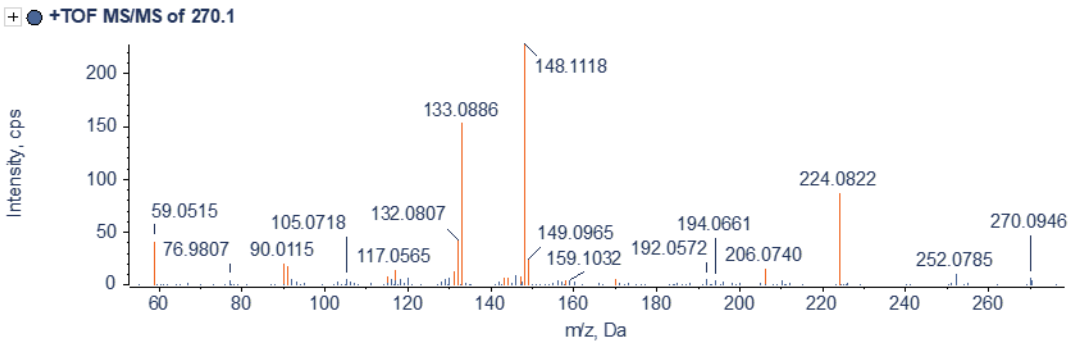

Figure S5. Secondary fragment ion of Acetochlor.

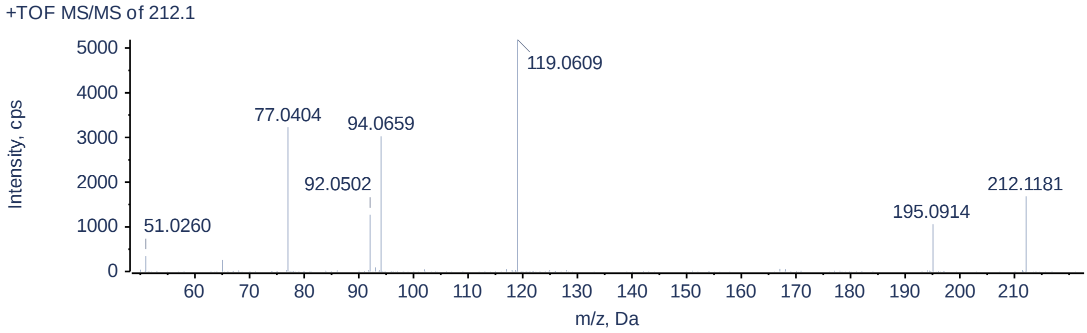

Figure S6. Secondary fragment ion of CMEPA.

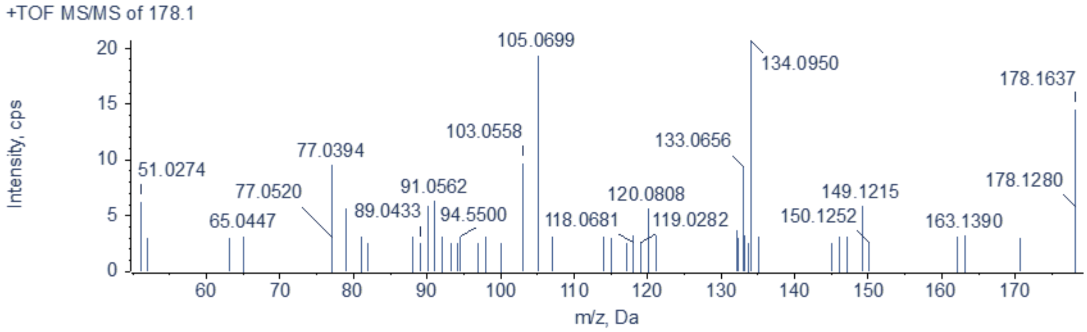

Figure S7. Secondary fragment ion of MEPA.

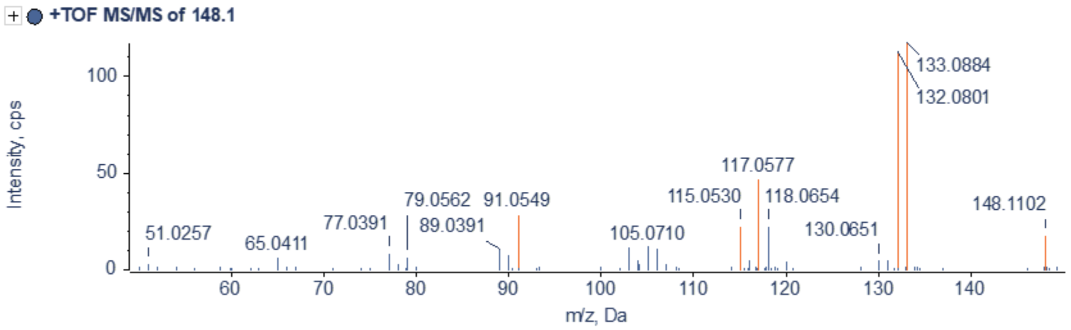

Figure S8. Secondary fragment ion of M147.

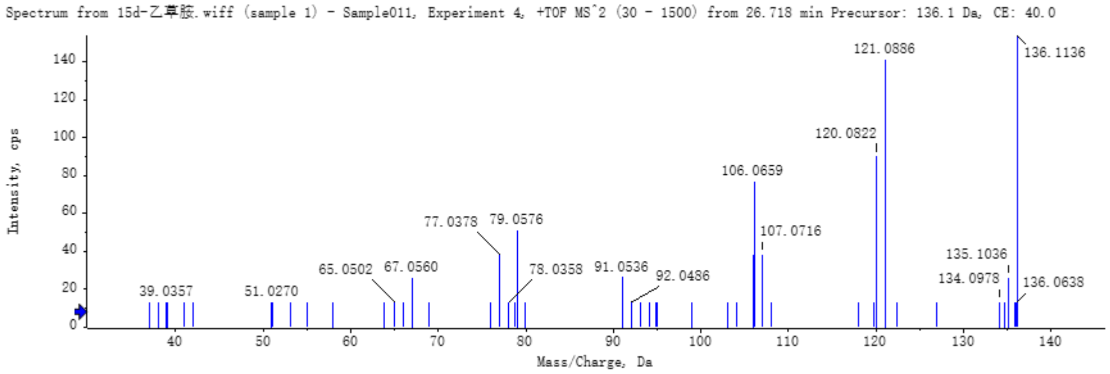

Figure S9. Secondary fragment ion of MEA.

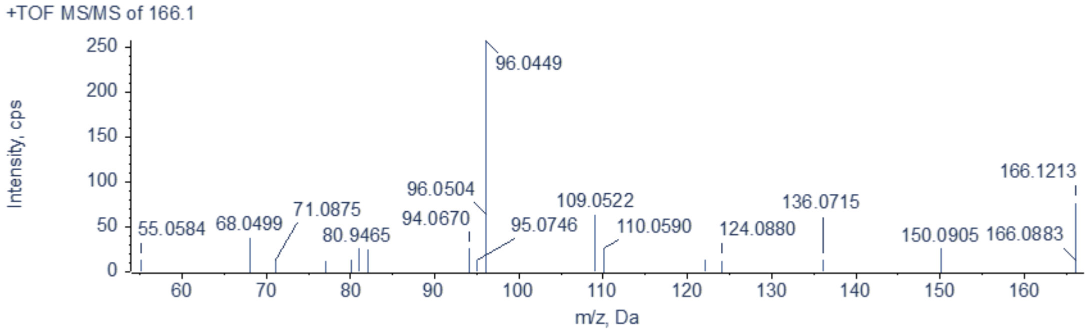

Figure S10. Secondary fragment ion of ECA.

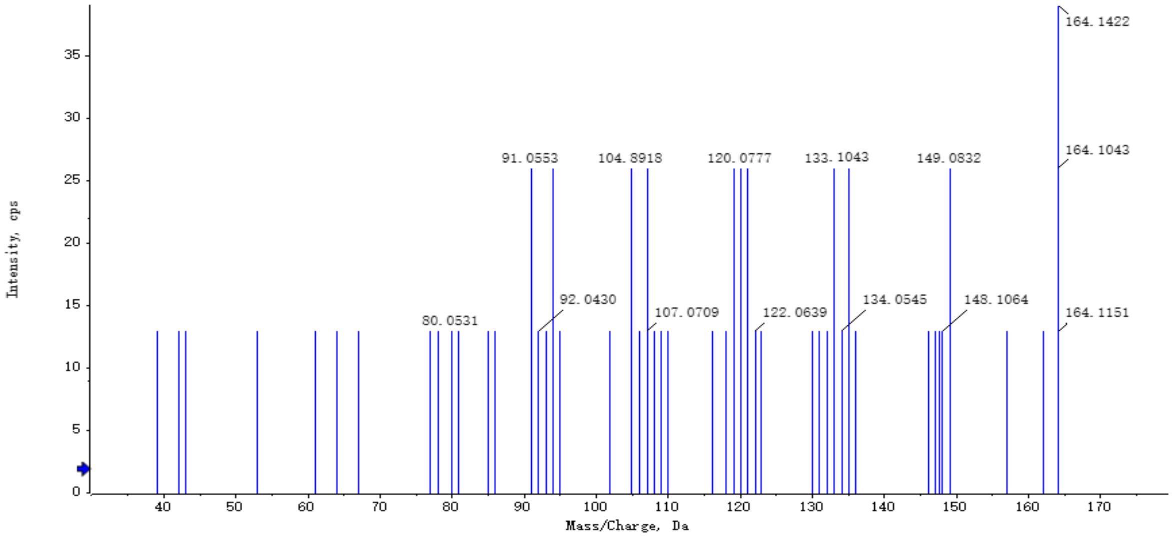

Figure S11. Secondary fragment ion of EPA.

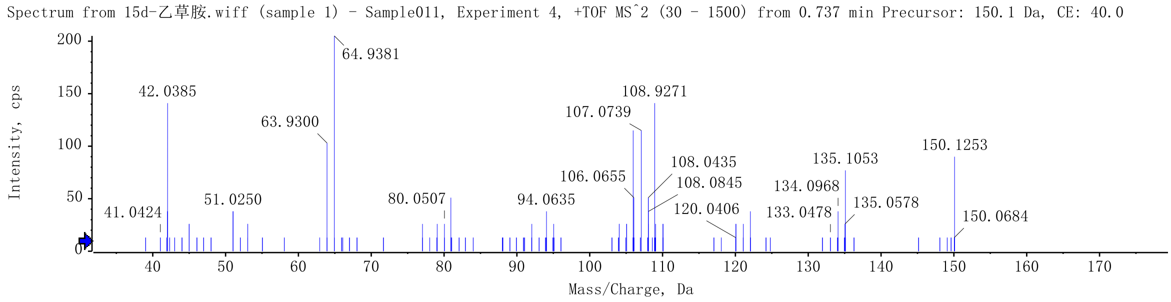

Figure S12. Secondary fragment ion of EPF.

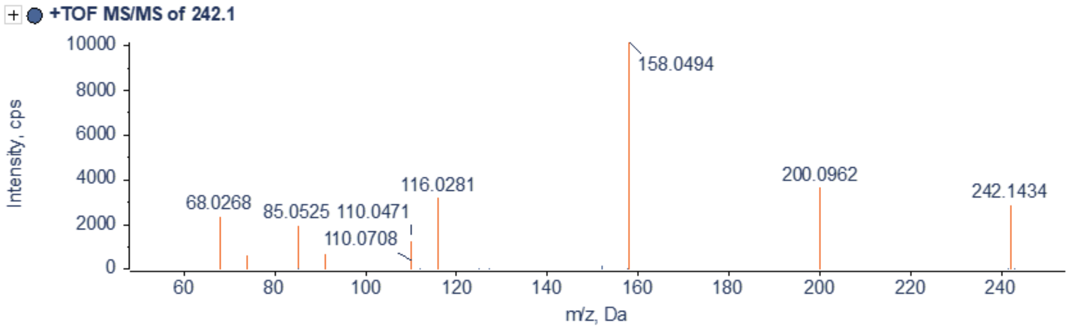

Figure S13. Secondary fragment ion of prometryn.

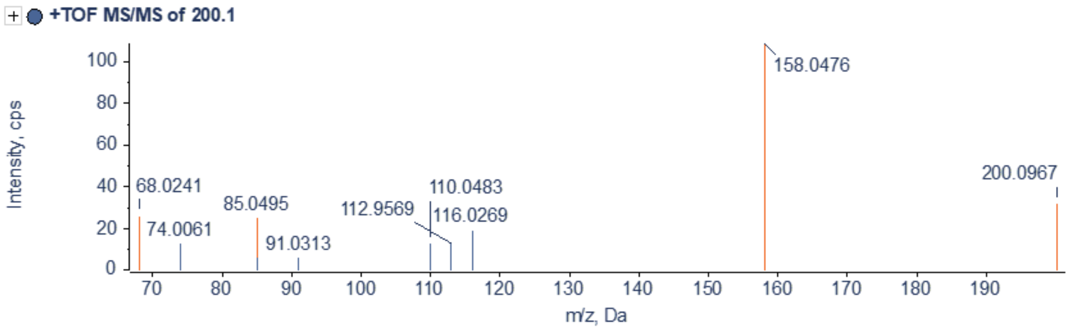

Figure S14. Secondary fragment ion of DMP.

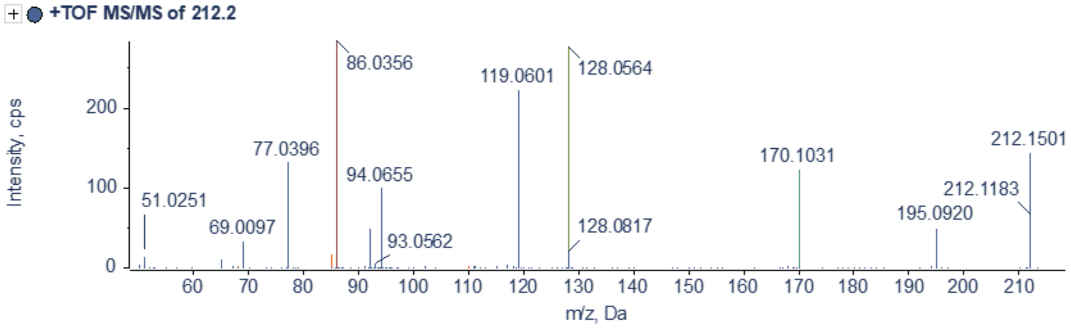

Figure S15. Secondary fragment ion of HP.

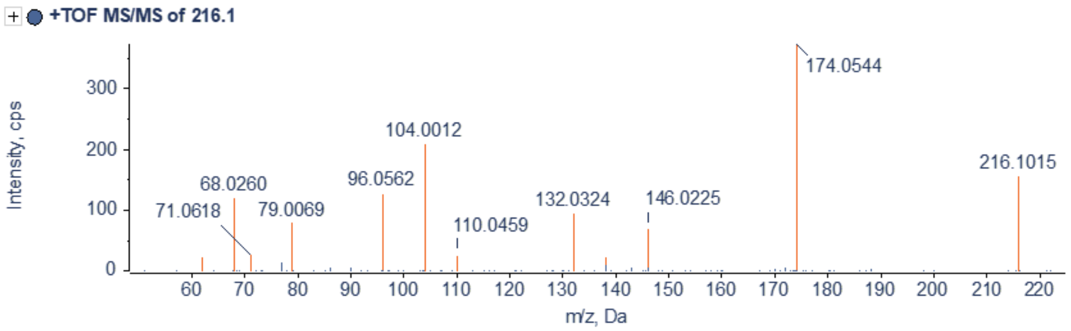

Figure S16. Secondary fragment ion of atrazine.

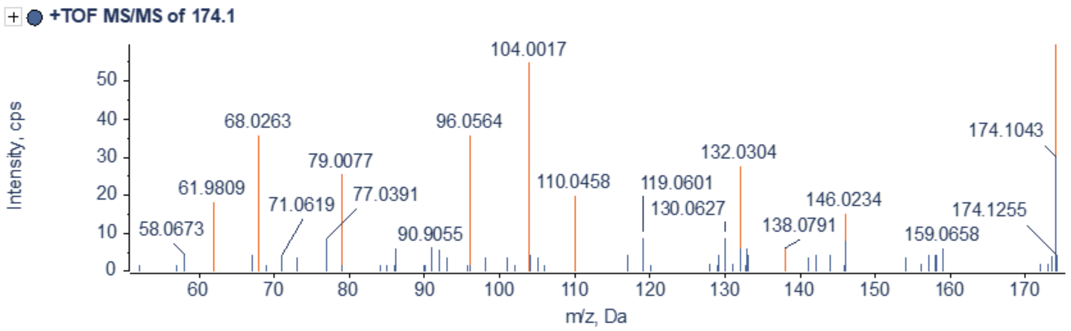

Figure S17. Secondary fragment ion of DIA.

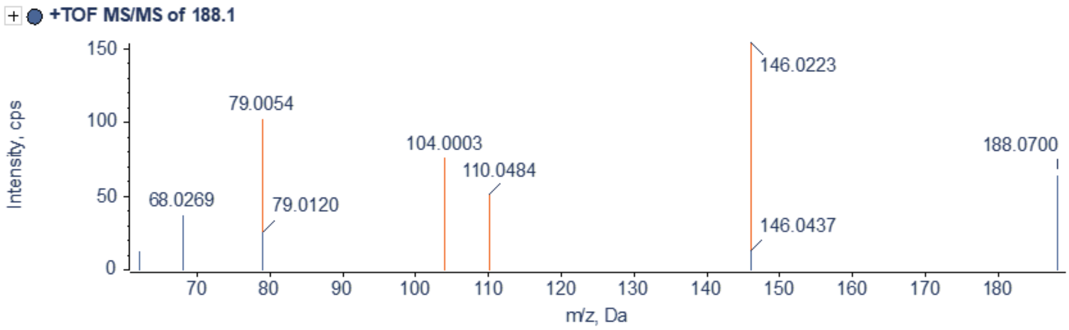

Figure S18. Secondary fragment ion of DEA.

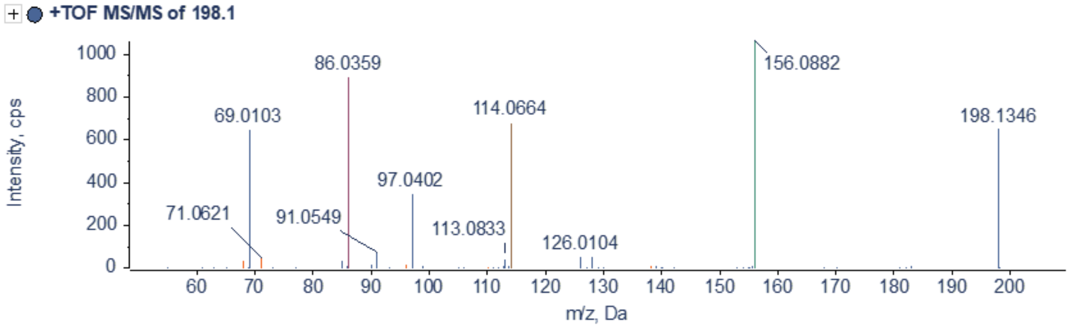

Figure S19. Secondary fragment ion of HA.
